# Body mass index and other anthropometric variables in children with sickle cell anaemia

**DOI:** 10.12669/pjms.322.9046

**Published:** 2016

**Authors:** Odutola Israel Odetunde, Josephat Maduabuchi Chinawa, Kingsley Ihedioha Achigbu, Eberechukwu O Achigbu

**Affiliations:** 1 Dr. Odutola Israel Odetunde, Pediatric Nephrology Unit, Department of Pediatrics, University of Nigeria Teaching Hospital, UNTH, Ituku Ozalla, Enugu, PMB 01129, Ituku - Ozalla, Enugu State, Nigeria; 2 Dr. Josephat Maduabuchi Chinawa, Pediatric Cardiology Unit, Department of Pediatrics, University of Nigeria Teaching Hospital, UNTH, Ituku Ozalla, Enugu, PMB 01129, Ituku - Ozalla, Enugu State, Nigeria; 3 Dr. Kingsley Ihedioha Achigbu, Department of Pediatric, Federal Medical Centre, Owerri, Imo State, Nigeria; 4 Dr. Eberechukwu O Achigbu, Department of Ophthalmology, Federal Medical Centre, Owerri, Imo State, Nigeria

**Keywords:** Anthropometry, Children, Sickle cell anaemia, Nigeria, Z-sore

## Abstract

**Objectives:**

The objectives of this study were to determine the anthropometric variables of children with sickle cell anaemia and comparing it with those with normal haemoglobin genotype.

**Methods:**

A cross sectional study of anthropometric measurements was conducted over a period of six months. Children with sickle cell anaemia in steady state aged between 6-20 years were recruited. Nutritional assessment was done using anthropometrical variables. Data were analyzed using the Statistical Package for Social Sciences program (SPSS), version 20.

**Results:**

The sickle cell patients comprised of 20 males and 20 females. There were an equal number of controls with an equal male to female ratio of 1:1. Forty eight percent (19) of the children with sickle cell anemia were underweight (< 5th %ile) and this is statistically significant. χ^2^=18.02 and p=0.000. When compared with subjects with normal haemoglobin genotype only five of them (13%) were underweight. χ^2^=10.286 and p=0.001. The controls weighed significantly more than the HbSS patients and also had significantly larger body surface compared to the HbSS population (P<0.05).

**Conclusion:**

BMI and other anthropometric variables among children with sickle cell anemia were low when compared with children with normal Haemoglobin genotype.

## INTRODUCTION

Sickle cell anaemia (SCA), a common inherited haematological disorder in Nigeria, presents with chronic haemolytic anaemia, musculoskeletal anomalies, recurrent infections and growth problems.^1^ Poor growth and nutrition are common in children with sickle cell anaemia (SCA), there exists an evidence of nutritional status in children with SCA in relation to anthropometric status, disease severity and body composition.^2^ For instance energy supply has been known to cause a constant perturbation in children with sickle cell anemia with its attendant effect on Body Mass Index (BMI). These disturbances could be due to increase demand from high metabolic rates, reduced absorption and increased degradation.^3^

Body composition changes significantly from infancy to adolescence in terms of absolute and relative changes in the amount of lipid, protein, water, and minerals, however this physiological changes may not be the same for children with sickle cell anemia.^3^,^4^

In sickle cell anemia, hypoxaemia and tissue hypoperfusion are the key notes. This as a result can cause impairment of tissue which then affects almost all systems of the human body causing retardation of growth and development. This is reflected in impairment in various anthropometric measurements e.g. Height, Weight, Body fat, skeletal maturation, delayed puberty.^4^,^5^

BMI is concerned with the measurement of the variation of the physical dimensions and the gross composition of the human body at different age levels.^6^,^7^ It is one of the important parameters for assessment of growth and development and nutritional status of the children.^6^,^7^

The monitoring of growth and nutritional status in children with SCA is a vital tool for comprehensive care, which facilitates early diagnosis of growth failure and nutritional intervention.^8^ Regrettably, the cut-off used for BMI classification is the same in both children with and children without sickle cell anaemia, yet there is a marked difference between body shape of children with sickle cell anaemia and that without sickle cell anaemia.^8^ In order to account for changes in this documented body shape, this work was carried out to find out if there is any change between these variables among children with sickle cell anemia and those with normal haemoglobin genotype.

## METHODS

### Study area

This study was carried out in the University of Nigeria Teaching Hospital (UNTH) Ituku-Ozalla Enugu. It is a tertiary health facility that services as a referral centre for various health centers in Enugu state and her environs.^9^

### Study population

These were patients with haemoglobin genotype HbSS attending the paediatric sickle cell clinic at UNTH Enugu, who fulfilled the criteria for inclusion into the study. The controls comprised healthy children with haemoglobin genotype HbAA matched for age and sex randomly selected from primary and post primary schools within Enugu metropolis.

### Ethical Consideration and Consent

Ethical clearance for the study was obtained from the Research and Ethical Committee of the University of Nigeria Teaching Hospital Ituku Ozalla. Informed consent was sought from parents or care givers.

### Study design

A cross sectional study of anthropometric measurements was conducted over a period of six months period. Children with sickle cell anaemia in steady state aged between 6-20 years with a consent obtained from the patient and parents/guardian were included in the study while children with postural deformity that affect the height were excluded from the study.

For the controls, the selection process was by simple random sampling. Weight in kilograms was taken with subjects in their school uniforms made of light fabric barefoot, holding onto nothing, using a normal bathroom standing scale [Deteco scales Inc. Brooklyn, New York, USA]. Recordings were made to the nearest 0.5kg.

Standing height in centimeters (cm) was measured with a stadiometer [CMS weighing equipment of 17 Campdem Road, London, NW1]. Measurements were without shoes, both feet flat on the ground and apposed at the medial malleoli. Recordings were made to the nearest 0.5cm.

Chest circumference was recorded in the horizontal plane through the fourth sternocostal junction at the end of normal expiration using a standard tape measure made of non irritant, non elastic material. Measurements were made to the nearest 0.5cm. Body surface area was computed with appropriate normogram.

The BMI was calculated by the formula: BMI = Weight (Kg)/height (M2). We used the World Health Organization and Centers for Disease Control to classify BMI into underweight as BMI less than the 5th percentile, healthy weight as BMI of 5th up to the 85th percentile, overweight as BMI of 85th to less than the 95th percentile and obese as BMI equal to or greater than the 95th percentile for age and gender.^7^,^8^

The social classes of subjects were determined using the mean of fathers’ occupation and mothers’ education. Socio-economic index scores were awarded to each child according to the method of Oyedeji.^10^

### Data Analysis

Data was analyzed using the Statistical Package for Social Sciences program (SPSS), version 20. Results are presented in cross tabulation and tables. Statistical tests were considered significant at a probability level of 5% (p value = 0.05).

## RESULTS

### Age and Sex Distribution

The distribution of the study population according to age and sex is illustrated in Table-I. The sickle cell patients comprised of 20 males and 20 females. There were an equal number of controls with an equal male to female ratio of (1:1). There was no significant sex or age difference between the HbSS patients and controls (p>0.05).

**Table-I:** Age and Sex Distribution of HbSS and Controls.

	Male		Female		Total	

Age groups (years)	HbSS	Control	HbSS	Controls	HbSS	Controls

	n (%)	n (%)	n (%)	n (%)	N (%)	N (%)
6-10	4(20.0)	4(20.0)	3(15.0)	3(15.0)	7(17.5)	7(17.5)
11-15	11(55.0)	12(60.0)	12(60.0)	12(60.0)	23(57.5)	24(60.0)
16-20	5(25.0)	4(20.0)	5(25.0)	5(25.0)	10(25.0)	9(22.5)

Total	20(100)	20(100)	20(100)	20(100)	140(100)	40(100)

X^2^ = 0.155 δf= 2 *p* > 0.05

### Socio- Economic Distribution

Majority of the HbSS patients and controls were in the low socio-economic group (class IV-V). This comprised of 60% of the HbSS patients and 57.5% of the controls. There were 27.5% of the HbSS patients and 25% of controls in the social class III while 12.5% of HbSS patients and 17.5% of controls respectively were in the elite or high social class (class I- II). The socio-economic spread of the HbSS patients compared to the controls were not statistically different (P>0.05).

BMI of children with sickle cell anemia when compared with subjects with normal haemoglobin genotype is shown in Table-II. Forty eight percent (19) of the children with sickle cell anemia were underweight (< 5th%ile) and this is statistically significant. χ^2^ =18.02 and p=0.000. When compared with subjects with normal haemoglobin genotype only five of them (13%) were underweight. χ^2^ = 10.286 and p=0.001. Male children (11) with sickle cell anemia had underweight malnutrition when compared with their female folk.

**Table-II:** Body Mass Index (BMI) distribution of the subjects (HbSS) and controls (HbAA) and Anthropometric Measurements of Subjects (HbSS) and Controls (HbAA).

Body Mass Index (BMI) distribution of the subjects (HbSS) and controls (HbAA)	Male	Female	Total	χ^2^	P value
Number of children assessed (Subjects – HbSS)	n = 19	n = 21	N = 40		
Underweight (< 5th %ile)	63%	33%	48%	18.029	0.000
Normal BMI (5th - 85th %ile)	37%	67%	53%		
Overweight or obese (≥ 85th %ile)*	0%	0%	0%		
Obese (≥ 95th %ile)	0%	0%	0%		
Total of subjects-HbSS	100%	100%	100%		
					
Number of children assessed (Controls- HbAA)	n = 20	n = 20	N = 40		
Underweight (< 5th %ile)	20%	5%	13%	10.286	0.001
Normal BMI (5th - 85th %ile)	80%	95%	88%		
Overweight or obese (≥ 85th %ile)*	0%	0%	0%		
Obese (≥ 95th %ile)	0%	0%	0%		

Total of control- HbAA	100%	100%	100%		

Anthropometric Measurements of Subjects (HbSS) and Controls (HbAA)					

Variables	HbSS (N=40)	Control (N=40)			

	Mean ± SD	Range	Mean ± SD	Range	P
Age (years)	12.3 ± 3.5	6 – 20	12.6 ± 3.5	6 – 17	> 0.05
Standing Height (cm)	145.9 ± 12.8	123 – 170	146.1 ± 12.9	122 – 170	> 0.05
Weight (kg)	34.4 ± 9.0	22.0 – 52.0	37.3 ± 10.4	19.5 – 61	< 0.05*
Chest Circumference (cm)	69.6 ± 6.5	57.0 – 86.0	70.2 ± 8.1	55.5 – 94	> 0.05
Body Surface Area (m^2^)	1.3 ± 0.2	0.94 – 1.70	1.3 ± 0.2	0.91 – 1.87	< 0.05*

Table-II also highlights a summary of age and anthropometric variables in the total study population. The mean age, standing height and chest circumference were comparable in both the HbSS patients and controls (P>0.05). The controls weighed significantly more than the HbSS patients and also had significantly larger body surface compared to the HbSS population (P<0.05).

Table-III summarizes the mean anthropometric measurements according to age groups in the HbSS patients and controls (total study population). It was observed that all the anthropometric variables increased consistently with age in both the HbSS patients and controls. Between 11-15 years of age weight and body surface area were significantly larger in the controls compared to the HbSS patients (p<0.05). Under 11 years of age (6-10years) the HbSS patients had larger mean weight, chest circumference and body surface area but smaller standing height compared to the controls. These differences in anthropometric measurements were only significant in chest circumference, with higher values in the HbSS patients.

**Table-III:** Anthropometric Measurements According to Age Groups for Subjects (HbSS) and Controls (Total Population) and Anthropometric Measurements According to Gender (Total Study Population).

Anthropometric Measurements According to Age Groups for Subjects (HbSS) and Controls (Total Population)									

Variables	6-10 years			11-15 years			16-20 years		

	HbSS (n=7)	Controls(n=7)		HbSS (n=24)	Controls (n=24)		HbSS (n=9)	Controls (n=9)	

	Mean ± SD	Mean ± SD	P	Mean ± SD	Mean ± SD	P	Mean ± SD	Mean ± SD	P
Standing Height (cm)	131.3 ± 6.4	131.6 ± 6.6	> 0.05	144.5 ± 10.0	144.6 ± 10.2	> 0.05	160.9 ± 6.8	161.3 ± 5.6	>0.05
Weight (kg)	25.3 ± 2.6	24.8 ± 5.0	> 0.05	33.0 ± 6.9	36.0 ± 7.0	< 0.05*	45.2 ± 6.6	50.6 ± 5.5	>0.05
Chest Circumference (cm)	62.2 ± 3.5	59.9 ± 3.0	< 0.05*	69.0 ± 4.8	70.6 ± 7.2	> 0.05	76.9 ± 4.7	77.1 ± 4.0	> 0.05
Body Surface Area (m^2^)	1.1 ± 0.1	1.1 ± 0.1	> 0.05	1.3 ± 0.2	1.3 ± 0.2	< 0.05*	1.6 ± 0.1	1.6 ± 0.1	> 0.05

Anthropometric Measurements According to Gender (Total Study Population)									

Variables				Male			Female		

				HbSS	Controls		HbSS	Controls	

				Mean ± SD	Mean ± SD	P	Mean ± SD	Mean ± SD	P
Standing Height (cm)				146.4 ± 14.5	146.2 ± 14.3	> 0.05	145.3 ± 11.7	145.6 ± 11.7	> 0.05
Weight (kg)				33.8 ± 8.4	36.5 ± 11.0	< 0.05*	35.0 ± 9.8	38.2 ± 10.1	< 0.05*
Chest circumference (cm)				68.7 ± 5.3	69.9 ± 9.1	> 0.05	70.4 ± 7.6	70.5 ± 7.1	> 0.05
Body Surface Area (m^2^)				1.3 ± 0.2	1.3 ± 0.3	< 0.05*	1.3 ± 0.2	1.3 ± 0.2	< 0.05*

Table-III also compares the anthropometric measurements in both sexes for the HbSS patients and controls. The male and female controls had generally higher values in all the anthropometric variables measured, compared to their HbSS counterparts. These differences were significant in weight and body surface area in both sexes (P<0.05). Height and chest circumference were comparable in the two groups though marginally higher in the control males and females. Males were taller than females among both control and HbSS populations but not significantly so (P>0.05). The females were heavier and had larger chest circumference and body surface area than the males in both the HbSS and control.

An analysis of the anthropometric variables according to age groups in the female population is summarized in Table-IV. All the anthropometric variables increased consistently with age in both groups of females. Between 6-10 years and 16-20 years of age, all the anthropometric variables were comparable in both the HbSS and control females (p>0.05). However between 11-15 years of age, the female controls were significantly heavier and had significantly larger body surface area compared to the HbSS females (p<0.05). Other anthropometric measurements were comparable in both groups of females at this age.

**Table-IV:** Anthropometric Measurements According to Age Groups and Gender for Subject (HbSS) and Controls (Females) and Anthropometric Measurements According to Age Groups and Gender for HbSS and Controls (Males).

Anthropometric Measurements According to Age Groups and Gender for Subject (HbSS) and Controls (Females)									

	6-10 years			11-15 years			16-20 years		

Variables	HbSS (n=7)	Controls(n=7)		HbSS (n=24)	Controls (n=24)		HbSS (n=9)	Controls (n=9)	

	Mean ± SD	Mean ± SD	P	Mean ± SD	Mean ± SD	P	Mean ± SD	Mean ± SD	P
Standing Height (cm)	131.0 ± 7.6	131.6 ± 7.6	> 0.05	142.7 ± 7.5	142.8 ± 7.5	> 0.05	160.2 ± 3.7	160.7 ± 3.5	>0.05
Weight (kg)	25.0 ± 3.0	24.3 ± 4.0	> 0.05	32.4 ± 7.5	36.9 ± 7.4	< 0.05*	47.2 ± 3.7	49.5 ± 3.8	>0.05
Chest Circumference (cm)	61.8 ± 2.3	59.5 ± 2.2	> 0.05	68.9 ± 5.6	70.5 ± 5.5	> 0.05	79.3 ± 4.6	77.1 ± 3.4	> 0.05
Body Surface Area (m^2^)	1.0 ± 0.1	1.0 ± 0.1	> 0.05	1.2 ± 0.2	1.3 ± 0.1	< 0.05*	1.6 ± 0.1	1.6 ± 0.1	> 0.05

Anthropometric Measurements According to Age Groups and Gender for HbSS and Controls (Males)									

	6-10 years			11-15 years			16-20 years		

Variables	HbSS (n=7)	Controls n=(7)		HbSS (n=24)	Controls (n=24)		HbSS (n=9)	Controls (n=9)	

	Mean ± SD	Mean ± SD	P	Mean ± SD	Mean ± SD	P	Mean ± SD	Mean ± SD	P
Standing Height (cm)	131.0 ± 6.6	131.6 ± 6.9	> 0.05	144.0 ± 10.7	144.0 ± 10.9	> 0.05	163.7 ± 4.5	164.0 ± 3.8	>0.05
Weight (kg)	25.0 ± 2.7	25.1 ± 6.2	> 0.05	31.3 ± 4.6	36.6 ± 7.1	< 0.05*	45.7 ± 3.7	49.8 ± 7.5	>0.05
Chest Circumference (cm)	62.5 ± 2.5	60.1 ± 3.9	< 0.05*	68.0 ± 3.0	70.7 ± 9.3	0 > 0.05	75.3 ± 1.9	75.9 ± 4.9	> 0.05
Body Surface Area (m^2^)	1.1 ± 0	1.1 ± 0.1	> 0.05	1.2 ± 0.2	1.3 ± 0.2	< 0.05*	1.6 ± 0	1.6 ± 0.1	> 0.05

Table-IV also shows an analysis of the anthropometric variables according to age groups in the male population. The HbSS males had significantly larger thoracic size (p<0.05) compared to the male controls between 6-10 years of age. From 11-15 years of age the male controls had significantly larger body surface area and also weighed significantly more than the HbSS males (p<0.05). Chest circumference and height were comparable in both groups of males at this age. All the anthropometric variables were comparable in both groups of males between 16-20 years of age though generally higher values were found in the male controls groups. These differences in weight, chest circumference and body surface area were not statistically significant (p>0.05).

## DISCUSSION

Consistent with our findings, where almost half of the children with sickle cell anemia were underweight with a male prepondearnace, other authors have also reported deficits in nutritional status among subjects with Sickle Cell Disease (SCD). For instance, Singhal et al.^11^ reported significantly lower weight, MUAC, and BMI in 20 Jamaican children with sickle cell anemia. This was also corroborated in one study in Nigeria which reported significant differences in weight, BMI, skinfold thicknesses, and MUAC among children with sickle cell anemia.^12^

We noted from this study, that male children with sickle cell anemia are underweight more than their female counterpart. It has been suggested that hormonal changes may have been associated with the different degree of growth retardation found among males because males with SCD often have severe hypogonadism and females with SCD have no comparable hormonal deficiency.^13^

When matched for age, the mean weight, standing height and body surface area were comparable in both the HbSS patients and controls below the age of 11 years (6-10 years). The HbSS patients however, were marginally heavier and had significantly larger chest circumference than the controls in this age group (6-10 years). Between 11-15 years of age, values of all measured anthropometric variables, were higher in the controls with significantly higher weight and body surface area compared to the HbSS patients.

After 15 years of age (16-20 years) values of the anthropometric variables of the HbSS patients again approached those of the controls giving comparable but marginally higher mean weight, height, chest circumference and body surface area in the control group.

Younger HbSS patients are likely to have experienced fewer episodes of crises compared to the older HbSS patients as well as the effect of such crises on their physical development. They may therefore have comparable physical development with matched controls with HbAA genotype as was the case in the present study between 6-10 years of age. For similar reasons, differences in anthropometric values between the HbSS patients and age matched controls, became more obvious with age resulting in significantly higher values in the controls between 11-15 years.

Between 16-20 years the difference in anthropometric values persisted with higher values in the controls compared to the HbSS patients. These differences however were not significant in this age group.

Previous authors have reported that height curves in male HbSS patients, approach that of healthy males with HbAA genotype by the age of 22 years and in females may actually exceed those of healthy females.^14^-^17^ It is possible that the narrowing of the differences in anthropometric values observed between the HbSS patients and matched controls after the age of 16 years may be a reflection of the early stages of such ‘catch-up’ growth.

It has been noted that HbSS patients tend to be slightly older than their height matched controls. This they believed is probably a result of delayed skeletal maturation observed in HbSS patients.^14^ In the present study, above the age of 16 years, most of the HbSS patients appeared to be slightly older than their height matched controls.. This slight difference in age may have further narrowed the gap in the values of anthropometric variables between the HbSS patients and controls resulting in marginally but not significantly higher values in the controls in this age group.

When the anthropometric variables were analyzed according to sex, the HbSS and control females were heavier and also had larger mean body surface area than their respective male counterparts. Other studies have documented similar findings, though different methodologies were used by them.^13^

A closer look at the female population in this study revealed that majority (60%) were aged between 11-15 years. This is noteworthy because, the hormonal changes of adolescence which is normally associated with general maturational changes and deposition of adipose tissue in females usually occurs maximally around this age.^18^ Similar adolescent changes in males have been found to occur at a later age.

When the total study population was analyzed according to sex and age, the controls had significantly higher values of anthropometric variables between 11-15 years compared to the SS patients in both sexes.

Delayed physical and sexual maturation is known to occur in HbSS patients.^19^ This delay in maturation is believed to result from the effect of chronic anaemia and low endocrine production, especially growth hormone in the HbSS patient in childhood. Such a delay in physical and sexual maturation in HbSS patients may explain the observed differences in the anthropometric variables in this age group.

## CONCLUSION

We noted from this study that BMI and other anthropometric variables among children with sickle cell anemia are low when compared with children with normal Haemoglobin genotype.
